# Synthesis of silver nanoparticles using white-rot fungus Anamorphous *Bjerkandera* sp. R1: influence of silver nitrate concentration and fungus growth time

**DOI:** 10.1038/s41598-021-82514-8

**Published:** 2021-02-15

**Authors:** Jerónimo Osorio-Echavarría, Juliana Osorio-Echavarría, Claudia Patricia Ossa-Orozco, Natalia Andrea Gómez-Vanegas

**Affiliations:** 1 Grupo de Bioprocesos, Departamento de Ingeniería Química, Facultad de Ingeniería, Universidad de Antioquia, Medellín, Colombia; 2 Grupo de Investigación en Biomateriales, Programa de Bioingeniería, Facultad de Ingeniería, Universidad de Antioquia, Medellín, Colombia

**Keywords:** Nanoparticles, Nanoparticles

## Abstract

Currently, silver nanoparticles (AgNPs) constitute an interesting field of study in medicine, catalysis, optics, among others. For this reason, it has been necessary to develop new methodologies that allow a more efficient production of AgNPs with better antimicrobial and biological properties. In this research growth time effects Anamorphous *Bjerkandera* sp. R1 and the silver nitrate (AgNO_3_) concentration over AgNPs synthesis were studied. Through the protocol used in this work, it was found that the action of the capping proteins on the surface of the mycelium played a determining role in the reduction of the Ag^+^ ion to Ag^0^ nanoparticles producing a particle size that oscillated between 10 and 100 nm. The progress of the reaction was monitored using visible UV–Vis spectroscopy and the synthesized AgNPs were characterized by scanning electron microscopy (SEM), transmission electron microscopy (TEM) and Fourier transform infrared radiation (FTIR) spectroscopy. The best synthetic properties were found at 1 mM of AgNO_3_ concentration, growth time of 8 days, and reaction time of 144 h. Nanometals obtention from microorganisms could be considered as a new method of synthesis, due to reducing abilities of metal ions through its enzymatic system and represents low-cost synthesis that reduces the generation of harmful toxic wastes.

## Introduction

Silver nanoparticles (AgNPs) have recently attracted considerable attention in the development of applications due to their excellent physical and chemical properties, such as its high thermal stability and low toxicity^1^. Studies have shown that these can overcome pathologies previously treated with conventional antibiotics, due to their strong antimicrobial characteristics and broad spectrum^2,3^. One of the challenges in terms of the synthesis process is to obtain nanoparticles with specific characteristics such as size distribution, shape, and surface charge, among others, that will in turn determine their physical and chemical properties^4^. The standardization of the nanoparticle synthesis process is a very important aspect since the antibacterial properties are highly related with their size and surface charge. If these properties are adequately controlled, silver nanoparticles could have an enormous potential as antibacterial agents^5,6^.

The methods mostly used for the synthesis of nanoparticles have been both physical and chemical^7^. The conventional physical methods tend to produce low nanoparticle quantities, while the chemical methods consume too much energy and require the use of stabilizing agents that are often toxic such as sodium dodecyl benzene sulfonate or polyvinylpyrrolidone (PVP), which are used to avoid nanoparticle agglomeration^8,9^. Therefore, there is a need to implement green and/or biological synthesis methods to reduce hazardous and toxic waste, with the possibility of obtaining particles on the nanometric scale^10^.

In the biological synthesis of AgNPs, the reducing and toxic stabilizing agents are replaced by nontoxic molecules (proteins, carbohydrates, antioxidants, etc.), produced by living organisms like bacteria, fungi, yeasts and plants^7,11^. For example, the implementation of fungi is considered an important synthesis route, due to the high binding capacity and intracellular metal uptake. It has been reported that fungal material is more advantageous with respect to bacteria and plants, being that the mesh-like fungal mycelium can withstand flow pressures, agitation and adverse conditions in processes that require the use of bioreactors and chambers^12^. Furthermore, fungi secrete significantly higher quantities of proteins than bacteria, which would amplify the productivity of nanoparticle synthesis^13^.

There are different fungi strains that have been studied to synthesize silver nanoparticles such as *Aspergillus niger, Aspergillus flavus, Alternaria alternate, Cladosporium cladosporioides, Fusarium solani, Fusarium oxysporum, Penicillium brevicompactum, Trichoderma asperellum and Verticillium*^14,15^. Azmath et al. for example identified that using culture filtrates from various *Colletotrichum *sp. synthesized AgNPs with sizes between 5 and 60 nm and found that the biomolecules secreted by the fungus possibly functioned as stabilizing agents to prevent them from agglomerating in the aqueous medium^16^. With respects to white-rot fungi^17–20^, their use has been reported in the biosynthesis due to their high tolerance to metals and their powerful enzyme system (protein release); this last property gives it a great capacity for adsorption of Ag^+^ ions on the walls of the mycelium^21^. Some white-rot fungi as *Phanarochaete chrysosporium*^19^, *Trametes ljubarskyi, Ganoderma enigmaticum*^18^ and *Trametes trogii*^22^ have been reported to produce stable silver nanoparticles when silver nitrate (AgNO_3_) is used as a metallic precursor in an aqueous medium, showing that fungal biomolecules under different experimental conditions play an important role in the production of AgNPs. Although many studies are known about the importance of using fungal material in obtaining nanoparticles, it is still necessary to evaluate some fungi as particle synthesizers and verify how their growth process affects the synthesis.

In the case of the fungus used in this study, it has been reported as an anamorphous of *Bjerkandera adusta*. These anamorphous are characterized by presenting asexual spores called conidia whose purpose is rapid reproduction and survival; this would mean great potential for various biotechnological and biomedical applications due to its high nutritional and organoleptic quality and the ease of growing on agro-industrial by-products^23^. The objective of this work was to evaluate the effect of silver nitrate concentration and growth time of fungus of on the synthesis of silver nanoparticles (AgNPs) from the white-rot fungus anamorphous *Bjerkandera* sp. R1. The formation of silver nanoparticles was monitored using UV–Vis spectrophotometry and complemented with its morphological characterization through scanning electron microscopy (SEM) and transmission (TEM).

## Methodology

### Microorganisms and culture media

White-rot fungus strain *Bjerkandera* sp. R1 was used and cryopreserved in pinewood splinters and bagasse. All fungi were donated by the Group of Environmental Biotechnology from the department of chemical engineering at Universidad de Santiago de Compostela (Spain)^24^. The reagents necessary for the preparation of the culture media were donated by the bioprocess group from the department of chemical engineering at Universidad de Antioquia (Colombia). Cultures were made every month in Petri dishes with solid Kimura medium [agar (15 g/L), glucose (20 g/L), peptone (5 g/L), yeast extract (2 g/L) KH_2_PO_4_ (1 g/L), MgSO_4_. 5H_2_O (0.5 g/L)] and pH 5.5^25^. The inoculum necessary to start all the assays were prepared by transferring 4 pieces of colonized agar to a Fernbach flask with liquid Kimura culture medium [glucose (20 g/L), peptone (5 g/L), yeast extract (2 g/L), KH_2_PO_4_ (1 g/L); MgSO_4_·5H_2_O (0,5 g/L)] and pH 5.5^25^. Subsequently, the mycelium layer formed was homogenized in a blender for 20 s for the different tests.

The crushed mycelium of fungus was mixed with Tween 80 and aseptically transferred to the liquid Kimura culture medium. The sample was incubated in a shaking incubator (JEIO TECH SI-300) at 30 °C at 200 rpm to favor the pellets formation; then it was centrifuged at 4500 rpm for 20 min to obtain two fractions: pellets and supernatant. Each of these fractions was then used to determine the effect of silver nitrate (AgNO_3_) concentration and growth time on the synthesis of silver nanoparticles.

### Evaluation of the operational conditions for the production of silver nanoparticles (AgNPs)

The production of AgNPs was carried out using two reduction methods.

1. Reduction of silver ions in the fungal filtrate (CS sample): 1% v/v solutions were prepared with the fungal filtrate obtained from the different growth time of fungus and the corresponding concentrations of AgNO_3_ and mixed for 144 h. Control for this sample was done only using the fungal filtrate.
2. Reduction of silver ions from the mycelium-pellets (MP sample): For this method the pellets in a 1% w/v concentration were mixed with the AgNO_3_ solutions and were incubated during 144 h, then the solution was centrifuged, and the pellets were separated by membrane filtration. Finally, they were re-suspended in deionized water and homogenized using a probe above 8.5 Hz for 5 min. Control of MP samples was carried out mixing the pellets (1% w/v) with aqua solution

#### Determination of a suitable AgNO_3_ concentration for the synthesis of AgNPs

To determine the influence of AgNO_3_ concentrations, the synthesis of AgNPs was evaluated using fungal filtrates from 5 and 7 days of growth because these times favored AgNPs formation according to the results previously reported by Osorio et al. (2014). For this reason, the fungal filtrate was mixed with three different final concentrations of AgNO_3_ (0.5, 1.0 y 1.5 Mm), they were incubated in the dark (30 °C) in a shaking incubator (JEIO TECH SI-300) at 200 rpm for 144 h. Each test was duplicated simultaneously for the AgNO_3_ solution, and a control was done only with the fungal filtrate. Samples were taken for 24, 48, 72, 96, 120 and 144 h (CS samples), and were analyzed using a UV–Vis spectrophotometer (Helios-α Thermo Spectronic) by scanning the absorbance spectra in 350–800 nm range of wavelength. The resulting spectra helped identify the absorption band of silver (Ag).

Table 1 shows the AgNO_3_ concentration values studied and the growth time of fungus on the response variable, area under the curve. The area under the curve of the UV–Vis spectra (AUC) was used because a quantitative variable was required to associate the presence or absence of AgNPs. The significance was determined using a variance analysis (ANOVA) and statistical program Statgraphics centurion ® was used for the response surface analysis.

**Table 1:** Attributes of factorial design multilevel CS samples of the fungus anamorphous *Bjerkandera* sp. R1.

Factors	Low	High	Levels	Units	Response variable
Growth time of fungus	5.0	7.0	2	Days	Area under the curve
AgNO_3_ Concentration	0.5	1.5	3	mM	

#### Evaluation of the effect of growth time of fungus on the synthesis of AgNPs

The most suitable AgNO_3_ concentration found in Sect. "Determination of a suitable AgNO3 concentration for the synthesis of AgNPs" was used to evaluate the effect of growth time of fungus. Six different growth days were evaluated for the fungus anamorphous *Bjerkandera* sp. R1 (3, 4, 5, 6, 7 and 8 days of culture). In this case, the CS samples was incubated in the dark (30 °C) in a shaking incubator (JEIO TECH SI-300) for 144 h at 200 rpm. These tests were carried out in triplicate. Additionally, a control was performed using only fungal filtrate, with the purpose of having a reference for the spectral analysis done. Small aliquots of CS samples were monitored every 24 h, for a total of 144 h by the scanning the absorbance spectra using a UV–Vis spectrophotometer (Helios-α Thermo Spectronic), with the same conditions as previously described.

Table 2 presents the studied values of growth time of fungus and incubation time, on the response variable area under the curve (AUC). The significance was determined using an analysis of variance (ANOVA) and the response surface analysis was done using the statistical program Statgraphics Centurion®.

**Table 2:** Attributes of factorial design multilevel CS samples of the fungus anamorphous *Bjerkandera* sp. R1.

Factors	Low	High	Levels	Units	Response variable
Growth time of fungus	3.0	8.0	6	days	Area under the curve
Incubation time	120.0	144.0	2	hours	

#### Characterization of the AgNPs

The evaluation of the size distribution of the AgNPs was performed by Transmission Electron Spectroscopy (TEM) and Scanning Electron Microscopy (SEM). For the SEM evaluation, the fungus was lyophilized, and small MP samples were fixed in a graphite tape. Additionally, a thin gold coating (Au) was placed (DENTON VACUUM Desk IV equipment) and analyzed in the scanning electron microscope (JEOL-JSM 6490 LV) with an accelerating voltage of 20 kV. Semi-quantitative chemical composition analysis of the sample was measured by energy dispersive X-ray Microscope-EDX (INCA PentaFETx3 Oxford Instruments) using the system coupled to the SEM equipment. For the TEM evaluation a Tecnai F20 Super Twin TMP instrument with an accelerating voltage of 200 kV and 0.1 nm resolution was used. For this, a drop of CS sample containing AgNPs was placed on a carbon coated copper grid, and samples were dried under an infrared (IR) lamp. A chemical compositional analysis of the colloidal suspension was measured by the detector EDX Oxford Instruments XMAX. The reported size distribution was found using calculated averages (10–20 measurements) over specific regions of the TEM and SEM micrographs. In this case, to measure the size of the nanoparticles both on the surface of the mycelium and in the colloidal suspension (CS samples), they were found using the internal software Scandium equipment for SEM and Image J for the TEM.

Finally, in order to determine the possible biomolecules responsible for the reduction of silver ions and for the confirmation of the capping agents on AgNPs, Fourier transform infrared radiation (FTIR) spectroscopy tests was performed (Nicolet iS50 FTIR). All measurements were carried out in the range of 400–4000 cm^−1^ at a resolution of 2 cm^−1^.

## Results and discussion

### Determination and influence of silver nitrate (AgNO_3_) concentration on the synthesis of AgNPs from the fungus anamorphous*** Bjerkandera*** sp. R1

#### Synthesis of silver nanoparticles (AgNPs) in the CS samples of the fungus anamorphous *Bjerkandera* sp. R1

**Figure 1:**
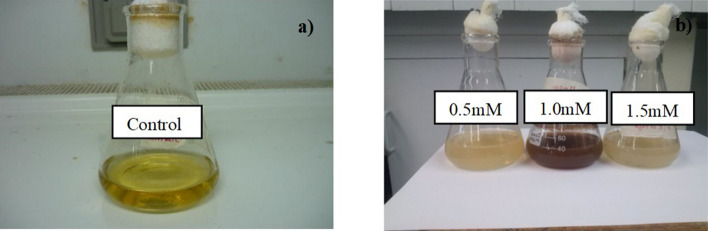
AgNPs biosynthesis in the CS sample (**a**) Control sample of the fungal filtrate of the fungus anamorphous *Bjerkandera* sp. R1; (**b**) Formation of silver nanoparticles after 48 h of incubation for different AgNO_3_ concentrations in the synthesis of AgNPs.

**Figure 2:**
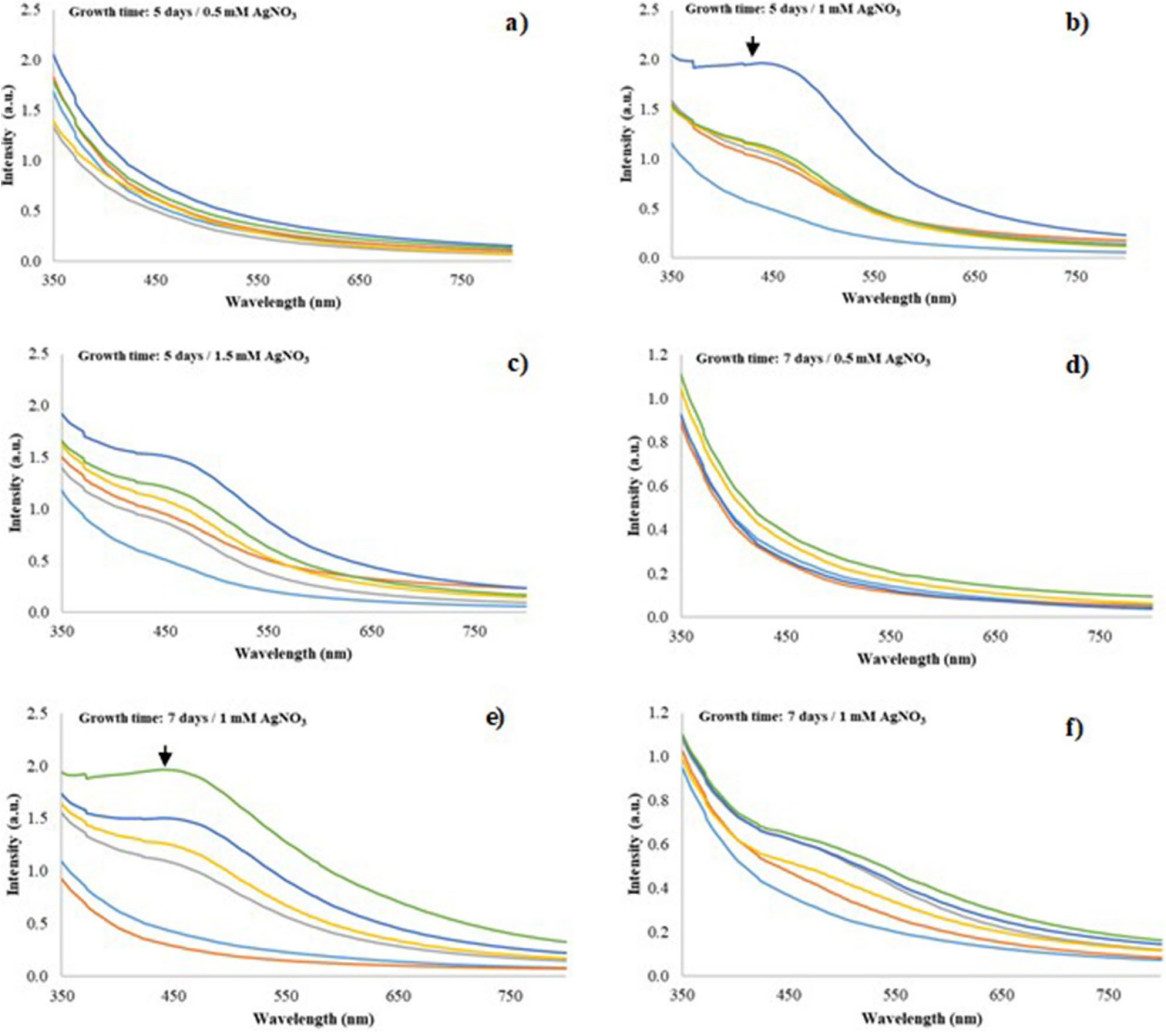
UV–Vis spectra for synthesized AgNPs using fungal filtrate from anamorphous *Bjerkandera* sp. R1 with a calculated error of: (**a**) Growth time: 5 days/0.5 mM AgNO_3_ (0.045–0.001); (**b**) Growth time: 5 days/1.0 mM AgNO_3_ (0.045–0.001), (**c**) Growth time: 5 days/1.5 mM AgNO_3_ (0.25–0.002); (**d**) Growth time: 7 days/0.5 mM AgNO_3_ (0.015–0.001); (**e**) Growth time: 7 days/1.0 mM AgNO_3_ (0.020–0.001) and (**f**) Growth time: 7 days/1.5 mM AgNO_3_ (0.4–0.010). Incubation time: 24 h  , 48 h  , 72 h  , 96 h  , 120 h  , 144 h  .

The reduction of the silver nanoparticles (AgNPs) in the fungal filtrate obtained from the white-rot fungus anamorphous *Bjerkandera* sp. R1 was examined through a qualitative analysis. A yellow to brown color change was observed after 48 h of reaction when the fungal filtrate was worked at final silver nitrate (AgNO_3_) concentrations of 1 and 1.5 mM respectively (Fig. 1). The color change explains the presence of AgNPs due to the Surface Plasmon Resonance (SPR) exhibited by the synthesized AgNPs^26,27^. With respect to the color change in these solutions, the determination of the size and shape of the synthesized AgNPs were initially corroborated through the absorption changes observed in the UV–Vis spectra (maximum wavelength) in function of time. A strong SPR at 430 nm was observed through the spectra, which increased its intensity with time and reached the stabilization point after 120 h for the fungal filtrate obtained from a growth time of fungus of 5 days and a concentration of 1 and 1.5 Mm AgNO_3_ (Fig. 2b,c). A broad SPR was also observed after 144 h for the fungal filtrate obtained from 7 days of growth of the fungus and concentration of 1.0 Mm AgNO_3_ (Fig. 2e), in contrast for a concentration of 1.5 mM AgNO_3_ a tenuous SPR was observed (Fig. 2f)_._ These changes in coloration, as well as the appearance of these bands were quantitative evidence of the presence of AgNPs in the solution.

Table 3 presents the ANOVA for an incubation time of 120 h and 144 h. According to the values obtained (*p* ≤ 0.05) with a confidence level of 95%, it was established that both the growth time of fungus variable and AgNO_3_ concentration showed no significant effect on the area under the curve of the UV–Vis spectra (AUC), for an incubation time of 144 h. In contrast, a significant effect was seen when the solution was incubated for 120 h.

**Table 3:** Variance analysis for the variable area under the curve.

Source	F-Ratio-120 h	*P* Value-120 h	F-Ratio-144 h	*P* Value-144 h
A:Growth time of fungus: 5 and 7 days	32.82	**0.0012**	0.14	0.7167
B:AgNO_3_ Concentration: 0.5. 1 and 1.5 mM	17.85	**0.0055**	1.17	0.3202
AB	0.21	0.6601	0.01	0.9390
BB	54.93	**0.0003**	6.03	**0.0494**

Growth time of fungus 120 and 144 h-anamorphous *Bjerkandera* sp. R1.

**Figure 3:**
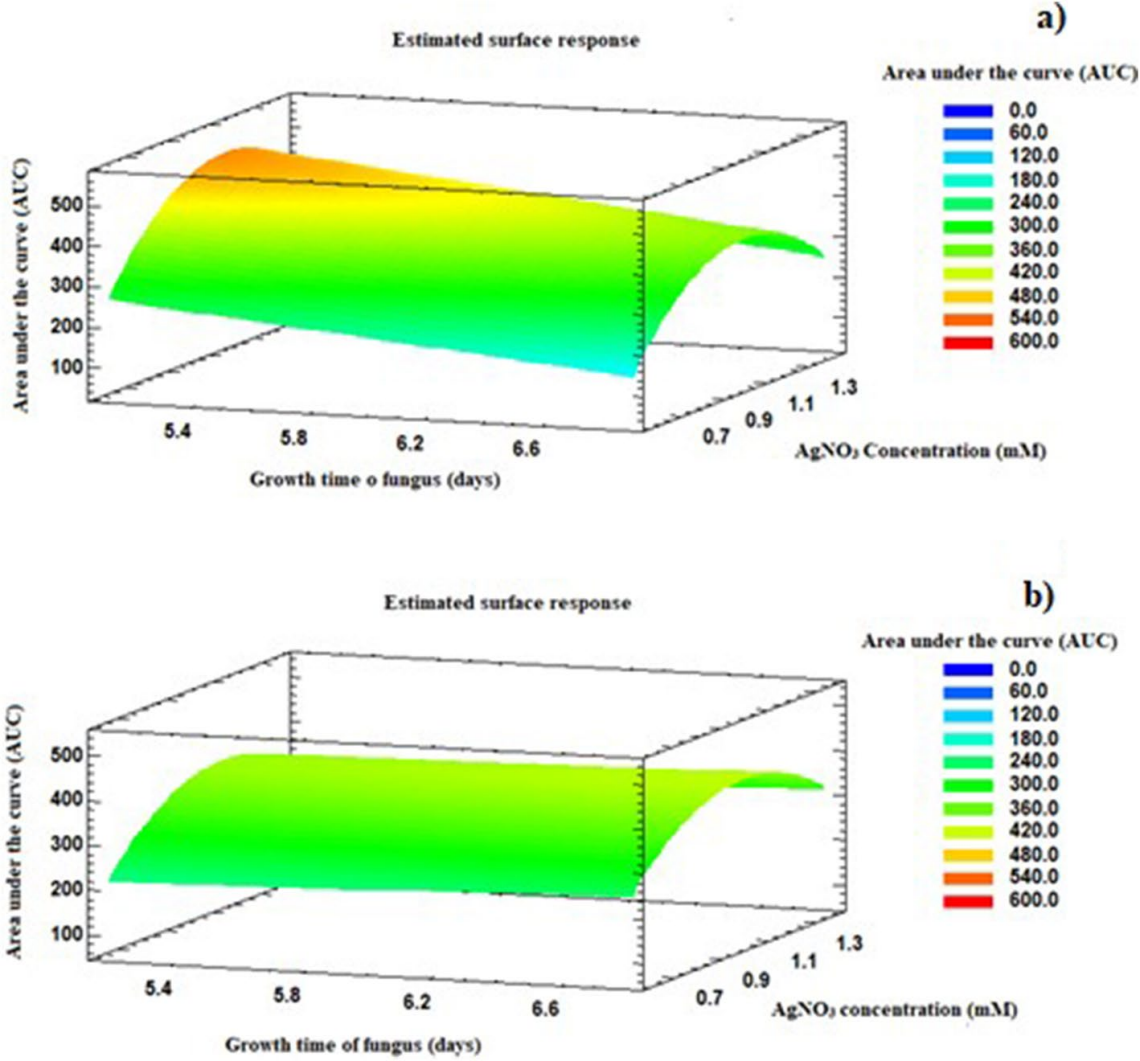
Response surface graphs for (**a**) t = 120 h (**b**) t = 144 h.

**Figure 4:**
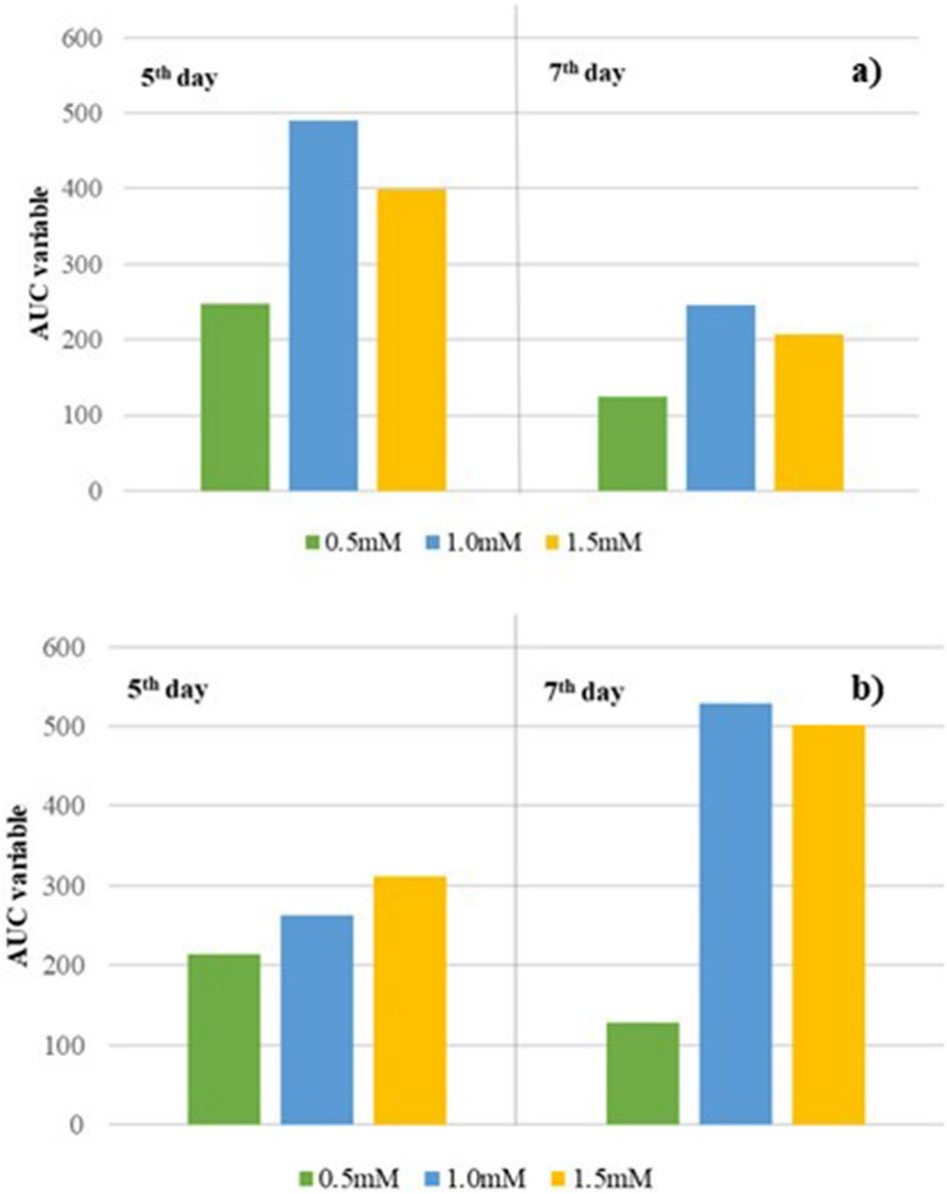
Area under the curve of the UV–Vis spectra (AUC). (**a**) t = 120 h (**b**) t = 144 h.

Figure 3 shows the response surface graphs on the AUC variable, after 120 and 144 h of incubation. A higher growth ‘rate’ favored an AUC increase and therefore the extracellular synthesis of AgNPs^28^. From these results, it was found that working with a AgNO_3_ concentration of 1 mM for 144 h was adequate for the synthesis of AgNPs using the fungus anamorphous *Bjerkandera* sp. R1. It was also found that the absorbance of these spectra increased and were higher in contrast to the solution worked at 1.5 mM (Fig. 4b). The absorbance of AgNPs (at the wavelength of maximal absorbance) is proportional to the concentration of AgNPs and these results indicated the formation of a greater number of AgNPs within the fungal extract (CS sample)^29^ and are in accordance with research conducted by Gudikandula et al.^18^ and Saravanan et al.^19^, who reported that working at a final concentration of 1 mM AgNO_3_ facilitates the stable formation of AgNPs from white-rot fungi.

#### Effect of silver nitrate (AgNO_3_) concentration on the biosynthesis of AgNPs using the fungus anamorphous Bjerkandera sp. R1

**Figure 5:**
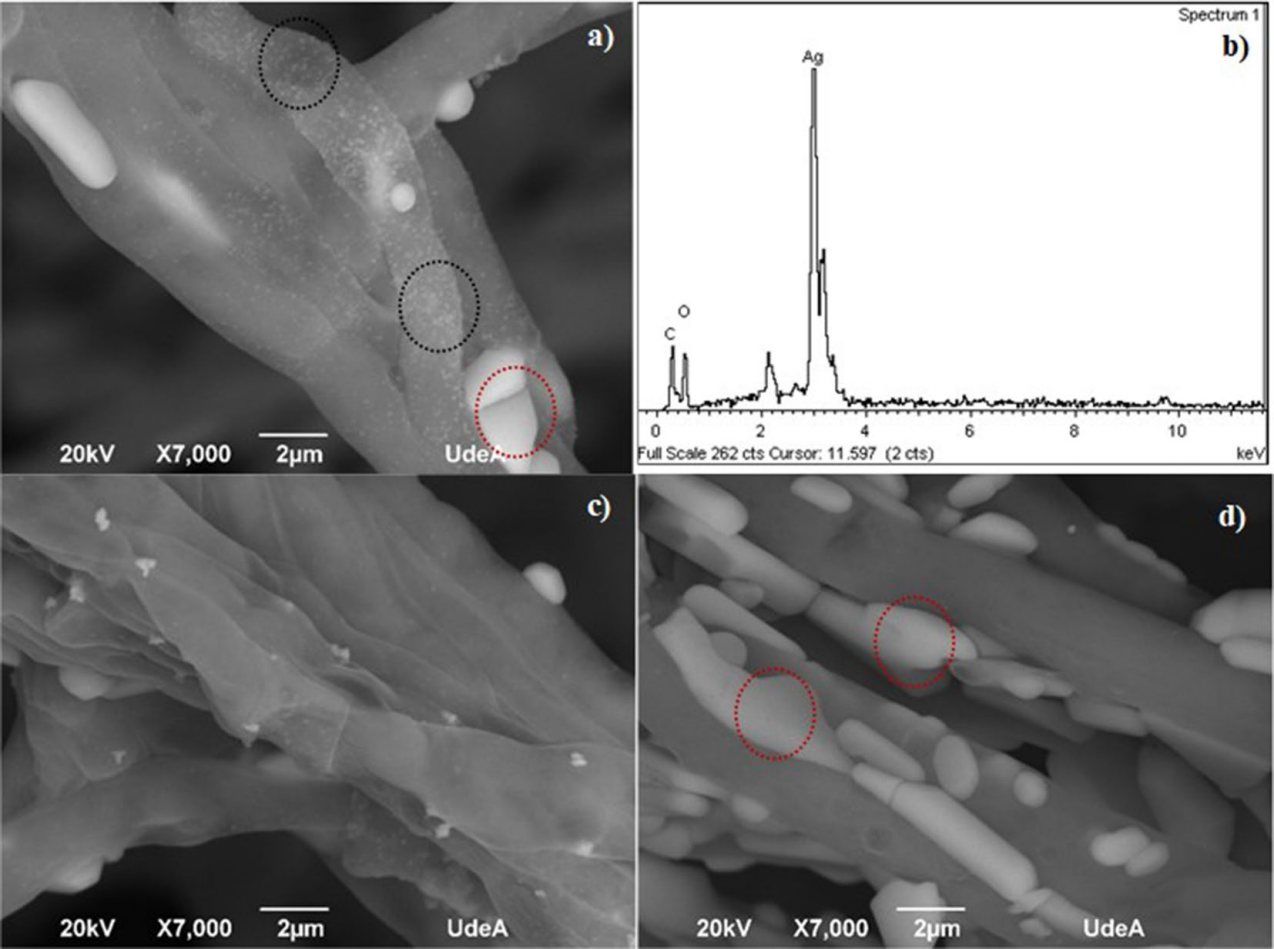
SEM–EDX analysis of the anamorphous *Bjerkandera* sp. R1 (**a**) 1 mM AgNO_3_ solution. Growth time: 7 days, incubation time 144 h. (**b**) EDX spectra 1 mM AgNO_3_ solution. Growth time: 7 days, incubation time 144 h (**c**) 0.5 mM AgNO_3_ solution. Growth time: 7 days. 144 h of incubation time (**d**) 1.5 mM AgNO_3_ solution. Growth time: 7 days. 144 h of incubation time.

To observe the effect of AgNO_3_ on the synthesis on the surface of the mycelium, measurements were taken using a scanning electron microscope (SEM). The SEM images show the micrographs of lyophilized fungus (MP samples) from day 7 of growth time of fungus (best result found according to Table 3, Fig. 3 and Fig. 4b on the CS sample), which was incubated for 144 h using different AgNO_3_ concentrations. Low accumulation of silver residues (macroparticles (Fig. 5a, see red circle) and well-defined particle distributions were observed for 1 mM AgNO_3_, with spherical shape and size distribution of 70–90 nm (Fig. 5a, see black circle). Regarding the other AgNO_3_ concentrations, the synthesis of AgNPs was highly regulated for a final concentration of 0.5 mM AgNO_3_ (Fig. 5b). In this case there was little formation of silver residues (Ag macroparticles)^17^ since the ions released in the solution were not adsorbed on the surface of the mycelium. From this analysis, this substrate concentration was not enough for some biomolecules to act appropriately as stabilizing and reducing agents. On the other hand, for a final concentration of 1.5 mM AgNO_3_ (Fig. 5c), the reduction of silver ions could have occurred intracellularly, but the combinations and interactions of the functional groups present in the wall of the fungus were affected by the implementation of higher levels of AgNO_3_; under these conditions the nucleation process of the Ag^+^ species became slower, which caused excessive accumulations of macroparticles on the surface of the mycelium (Fig. 5c, see red circle). Regarding the ideal concentration for intracellular synthesis, the results found are different from those reported by Kobashigawa et al. where it was found that 5 mM AgNO_3_ favors both intra and extracellular synthesis of AgNPs from white-rot fungus *Trametes trogii*^22^. In contrast to the fungus implemented in this research, the EDX spectra revealed the synthesis of AgNPs, due to the presence of a peak at approximately 3 keV that corresponds to the formation of pure silver^14,30^, indicating that a final concentration of 1 mM is ideal for carrying out the reduction of the Ag^+^ ion to Ag^0^ from the fungus anamorphous *Bjerkandera* sp. R1. In the EDX support carbon and oxygen peaks can also be observed, these peaks could indicate the presence of proteins and/or fungal filtrate remains that were retained in the interstitial spaces of the fungus. As described in the methodology, the culture medium is rich in carbon and this is an essential element that the fungus requires to fulfill its metabolic functions^24,31^.

### Effects of growth time of the fungus anamorphous *Bjerkandera* sp. R1 on the synthesis of AgNPs

#### Synthesis and characterization of AgNPs of the CS samples using the fungus anamorphous *Bjerkandera* sp. R1

**Figure 6:**
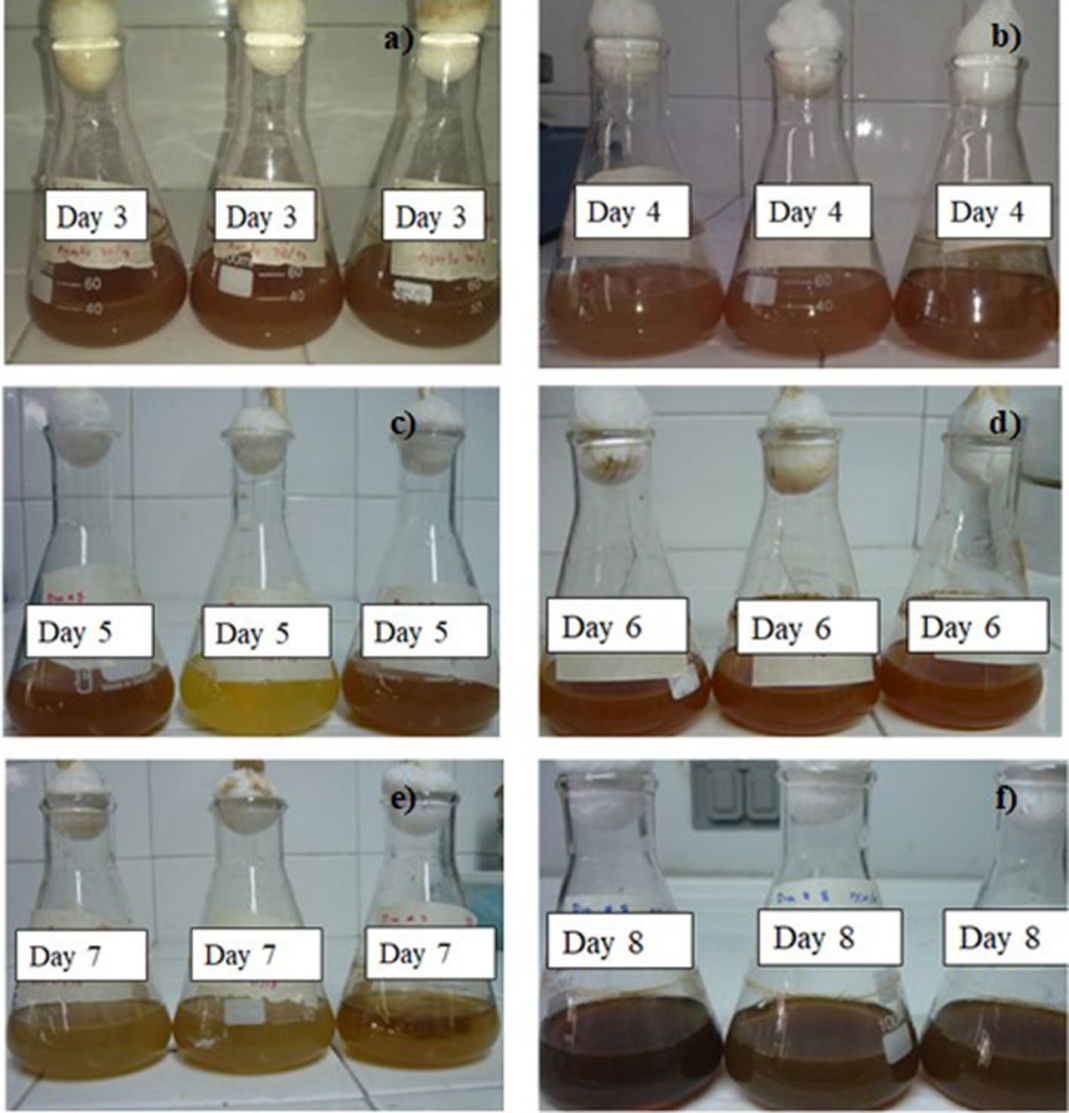
Formation of AgNPs after (**a**) 48 h of being incubated using a concentration of 1 Mm AgNO_3_ for different growth times of the fungus anamorphous *Bjerkandera* sp. R1.

Regarding the influence of growth time of fungus on the synthesis of AgNPs, a change of color was evidenced with respect to the positive control for all the CS samples from all the different growth times of the fungus (Fig. 6). This sharper contrast in color elucidates a higher proportion of AgNPs due to the surface plasmon resonance (SPR)^26,27^.

**Figure 7:**
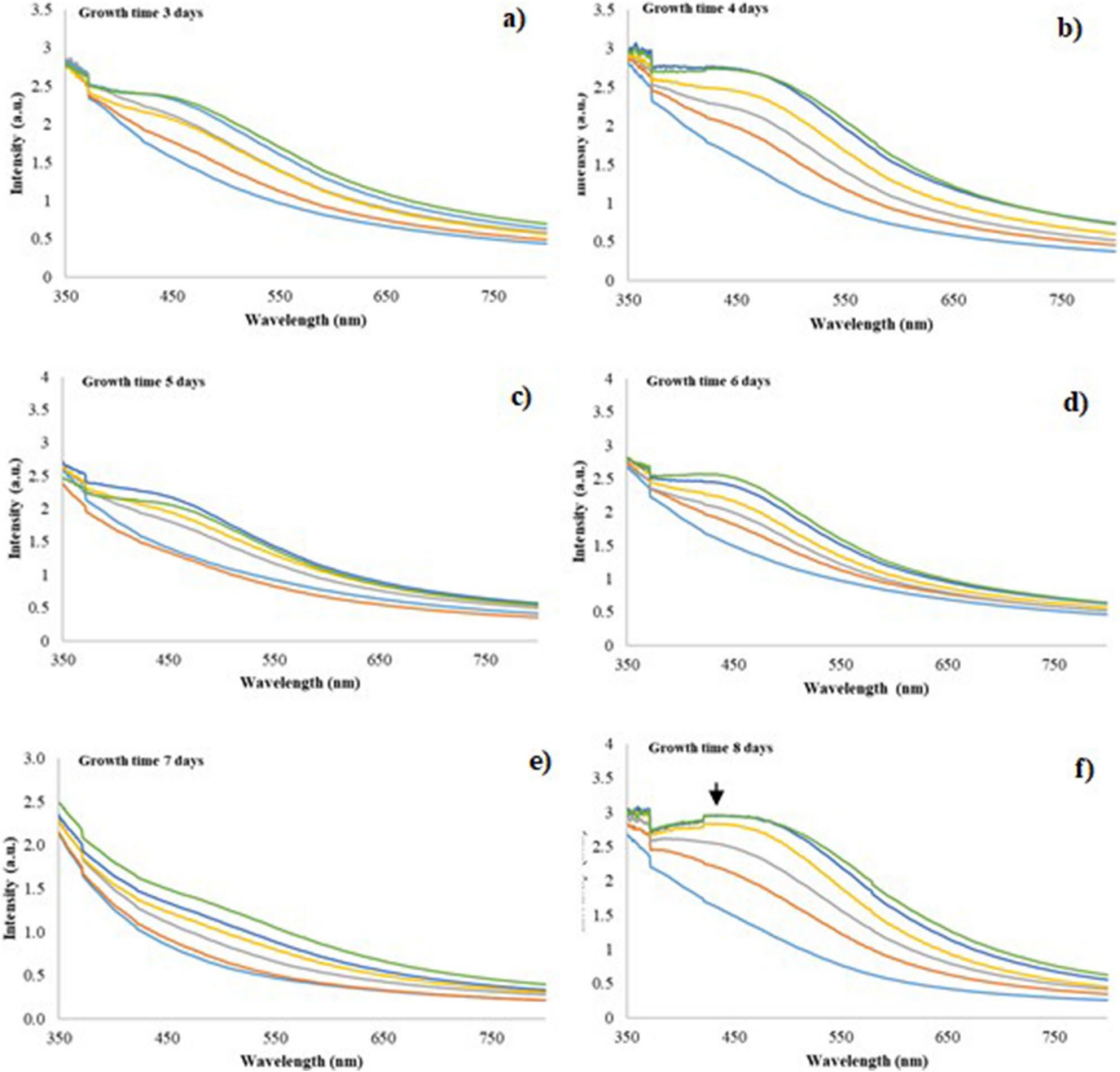
UV–Vis spectra for synthesized AgNPs using fungal filtrate from anamorphous *Bjerkandera* sp. R1 with a calculated error of: (**a**) Growth time 3 days (0.020–0.001); (**b**) Growth time 4 days (0.10–0.02); (**c**) Growth time: 5 days (0.15–0.004); (d**)** Growth time: 6 days (0.1–0.001) (**e**) Growth time:7 days (0.04–0.0008) and f) Growth time: 8 days (0.04–0.001). For a concentration of 1 mM AgNO_3_. Incubation time: 24 h  , 48 h  , 72 h  , 96 h  , 120 h  , 144 h  .

The change in color (CS samples in triplicate) were also verified with the Uv–Vis spectra. The higher absorbance peaks were seen at 430 nm for the fungal extracts obtained from the following growth days: 4, 5, 6, 7 and 8 (Fig. 7). For the latter growth day, the highest absorbance band was seen after 144 h of reaction with 1 mM AgNO_3_ (CS sample) (Fig. 7f). With respect to day 7 of growth, it can be established that the filtered fungal extract from this day, at the time of reacting with AgNO_3_ 1 mM, showed much less color compared to day 6 and 8. This probably could have occurred because under these conditions anamorphous *Bjerkandera* sp. R1 finished its stationary growth phase and entered the death phase. In this case, the secretion of proteins involved in the stabilization and reduction process of Ag^+^ ions could be affected, causing a low synthesis rate in the fungal extract and an adverse effect regarding the dispersion of the synthesized AgNPs^23^. With these growth days evaluated for the synthesis, it could be seen that there were no shifts to the left (blue) or to the right (red) on the maximum wavelength in the SPR peak; this process indicated according to Mie's theory^32^, that the anisotropy of the AgNPs decreased considerably and that the size could have possibly been controlled (Fig. 7a-e)^32–34^. In this study, the SPR bands founds suggested that the nanoparticles synthesized were spherical^32^.

**Figure 8:**
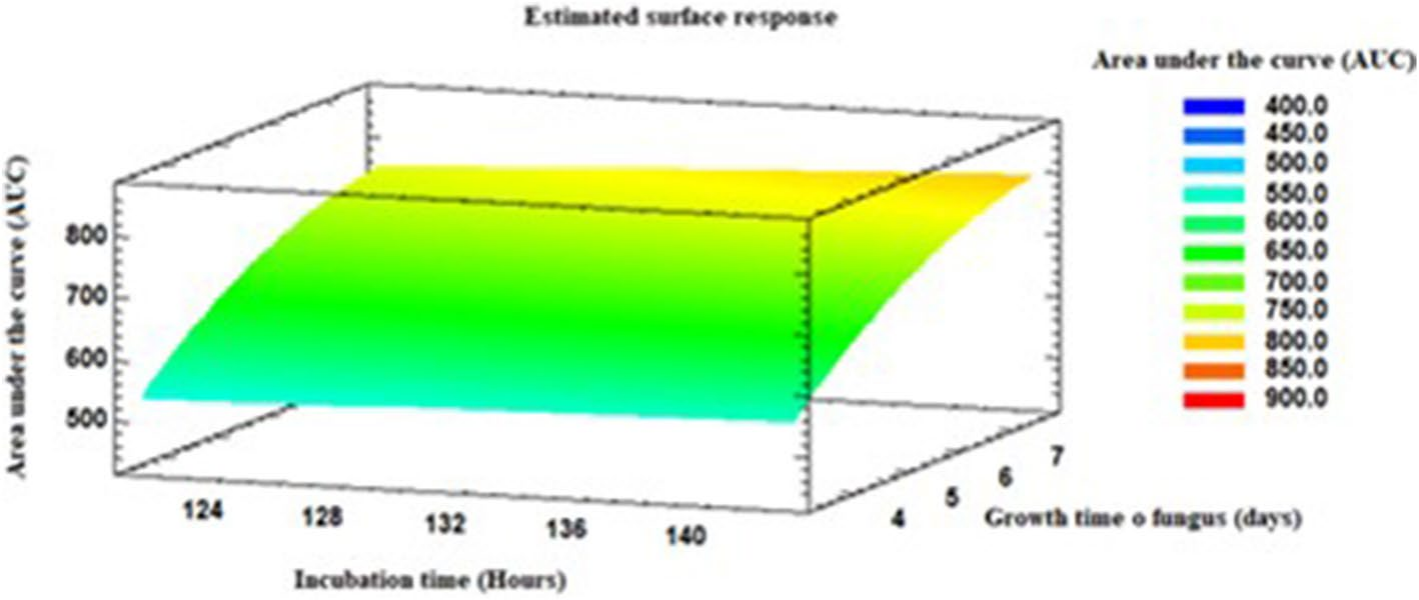
Response surface graphs. Effect growth time of the fungus in the synthesis of AgNPs.

Table 4 presents the ANOVA on the effects of growth time of fungus, according to the values achieved (*p* ≤ 0.05) with a confidence level of 95%. It was established that the growth time of the fungus had a significant effect on the response variable, area under the curve of the UV–Vis spectra (AUC). Observing the response surface graph, the best growth results obtained for the fungal extract was seen on the 8th day, which was incubated for 144 h with 1 mM AgNO_3_ (Fig. 8). With regards to this result, the optimal time required for the fungus to release more biomolecules in charge of the reduction process is the 8th day, and a greater AgNPs production is more feasible with longer reaction times with 1 mM AgNO_3_. These findings can be compared with the research carried out by Birla et al. where it reported that an absorbance peak intensity increase over time indicates the continuous reduction of silver ions and an increase in the concentration of AgNPs^35^.

**Table 4:** Analysis of variance for the variable area under the curve.

Source	F-Ratio	*P* Value
A: Growth time of fungus	19.38	**0.0001**
B: Incubation time	0.64	0.4286
AA	1.00	0.3259
AB	0.05	0.8177

Effect growth time of fungus in the synthesis of AgNPs.

**Figure 9:**
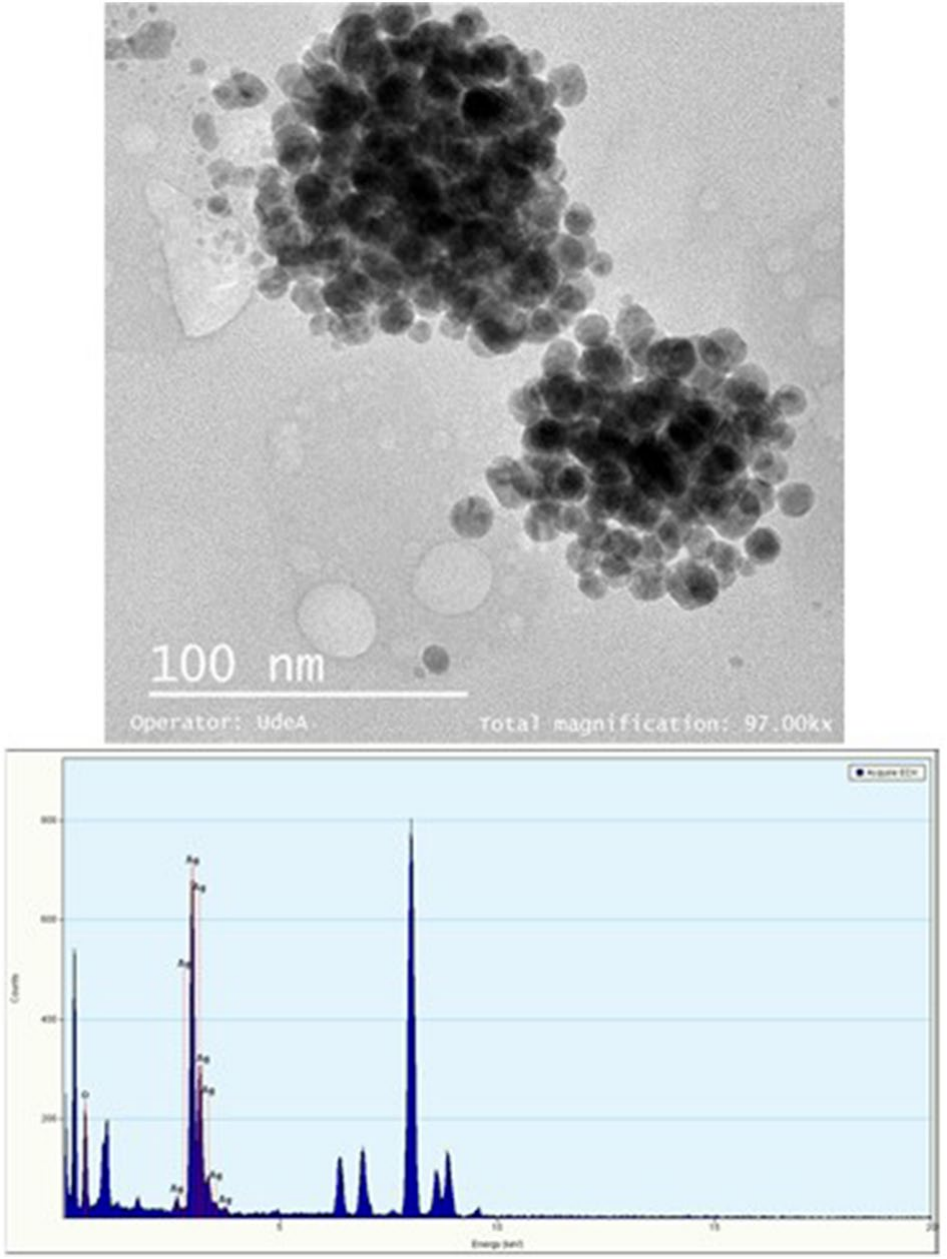
Transmission Electron Microscopy (TEM) images and EDX spectra of silver nanoparticles synthesized in the CS samples of the fungus anamorphous *Bjerkandera* sp. R1. Growth time of fungus: 8 days. Incubation time: 144 h.

Considering the previously cited results, the TEM micrographs (Fig. 9) show the difference in size and shape of the AgNPs once the fungal filtering was adjusted under the different conditions worked with. Most of the particles observed were spherical and separated from each other, with little agglomeration and size distribution between 10 and 30 nm. These results suggest that in this process, biological residues (capping agents) may have performed the function of reduction and stabilization of AgNPs^36,37^, as reported by Seetharaman et al.^14^, Saravanan et al. ^19^ and Balakumaran et al.^38^ in studies using different types of fungi. Analysis through Energy Dispersive X-ray (EDX) confirmed the presence of elemental silver signal (Fig. 8). Identification lines for the major emission energies for silver (Ag) are displayed in a range between 2.8 and 3.4 keV confirming the presence of AgNPs in the fungal filtrate. Other peaks appear in the EDX spectra; this indicates that in the process biomolecules were bound to the surface of AgNPs^39^.

#### Effect of growth time on the biosynthesis of AgNPs using the fungus anamorphous *Bjerkandera* sp. R1

**Figure 10:**
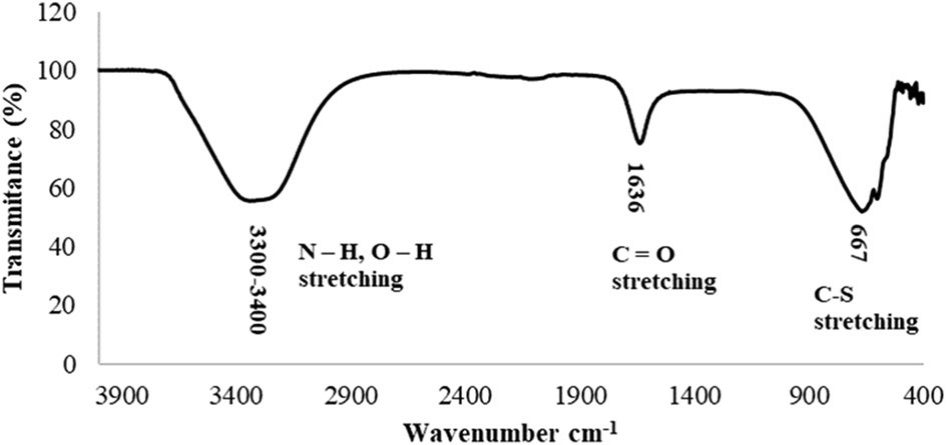
FT-IR spectra of AgNPs synthesized by anamorphous *Bjerkandera* sp. R1.

To evaluate the possible molecules involved in the process of reduction and subsequent formation of AgNPs FTIR was used (Fig. 10). These analyzes showed a broadband between 3300 and 3500 cm^−1^. This band can be assigned to the –N–H stretching vibrations of amide band I and the O–H stretching of aromatic amines. On the other hand, a prominent band was found between 1630 and 1680 cm^−1^, this band appears due to the vibrations of the –C=O (carbonyl) stretching vibrations in the amide bond of the proteins secreted by the fungus in the CS sample^29,40^. Another band appeared at 667 cm^−1^, this band corresponds to C–S stretching vibrations and possibly corresponds to heterocyclic compounds that can be found in the fungal filtrate^41^. From these results it was shown that probably the strong functional groups like carbonyl (–C=O) present in the fungal filtrate had more participation in the synthesis. In this context, FTIR study confirmed that possibly the adsorptive carbonyl group from amino acid residues and peptides of proteins had the stronger ability to bind silver ions (Ag^+^) in the mycelium^21^, and then; the bioreduction of these ions both the wall of the fungus and the CS sample was perhaps due to the release of some proteins which managed the nucleation and subsequent synthesis of AgNPs^15,40,42,43^, for this conditions, an adequate affinity between the substrate and the biomolecules responsible for the reduction process may have been achieved^26^. These findings are in agreement with several investigations that argue that functional groups present in the extracellular substances work as a capping agent and better adsorb the particles located on the surface of the mycelium, sealing the AgNPs and forming a coating with that prevents them from agglomerating when the sample is being rocked^17,40,44^.

**Figure 11:**
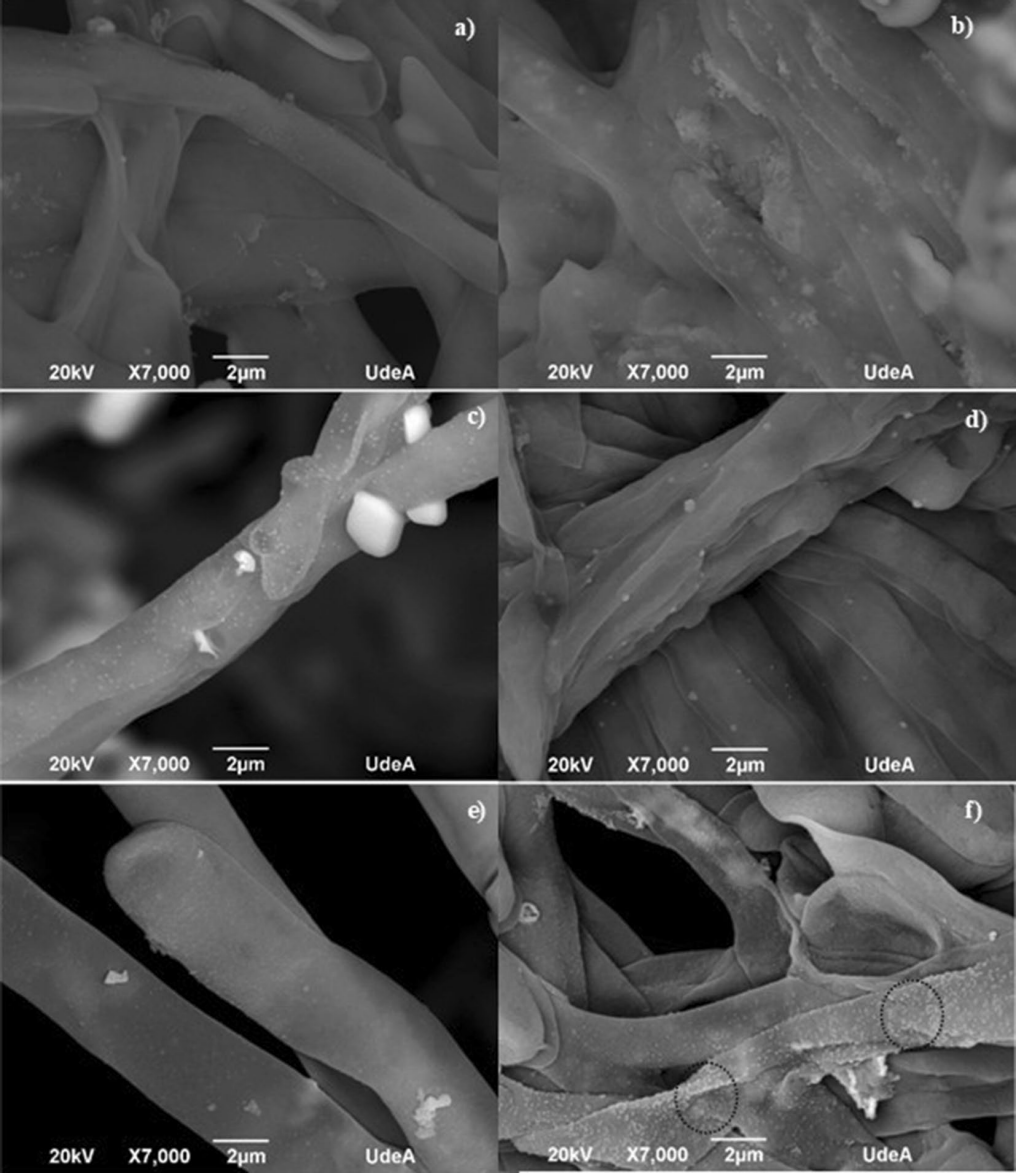
SEM micrographs of the fungal mycelium anamorphous *Bjerkandera* sp. R1 treated with a 1 mM AgNO_3_ solution. Growth time. (**a**) 3 days. (**b**) 4 days. (**c**) 5 days. (**d**) 6 days (**e**) 7 days and (**f**) 8 days.

According to the evaluated days of growth, the SEM images show that most of the AgNPs biosynthesized, present on the cell surface, are spherical with an approximate size of 30–100 nm. The result indicated that the reduction process occurred on the surface of the mycelium and that the AgNPs were uniformly distributed, forming small groups for the fungal material for all the growth times (Fig. 11a–e); but inhibiting the agglomerate formation for the fungus that grew for 8 days (Fig. 11f, see black circles).

**Figure 12:**
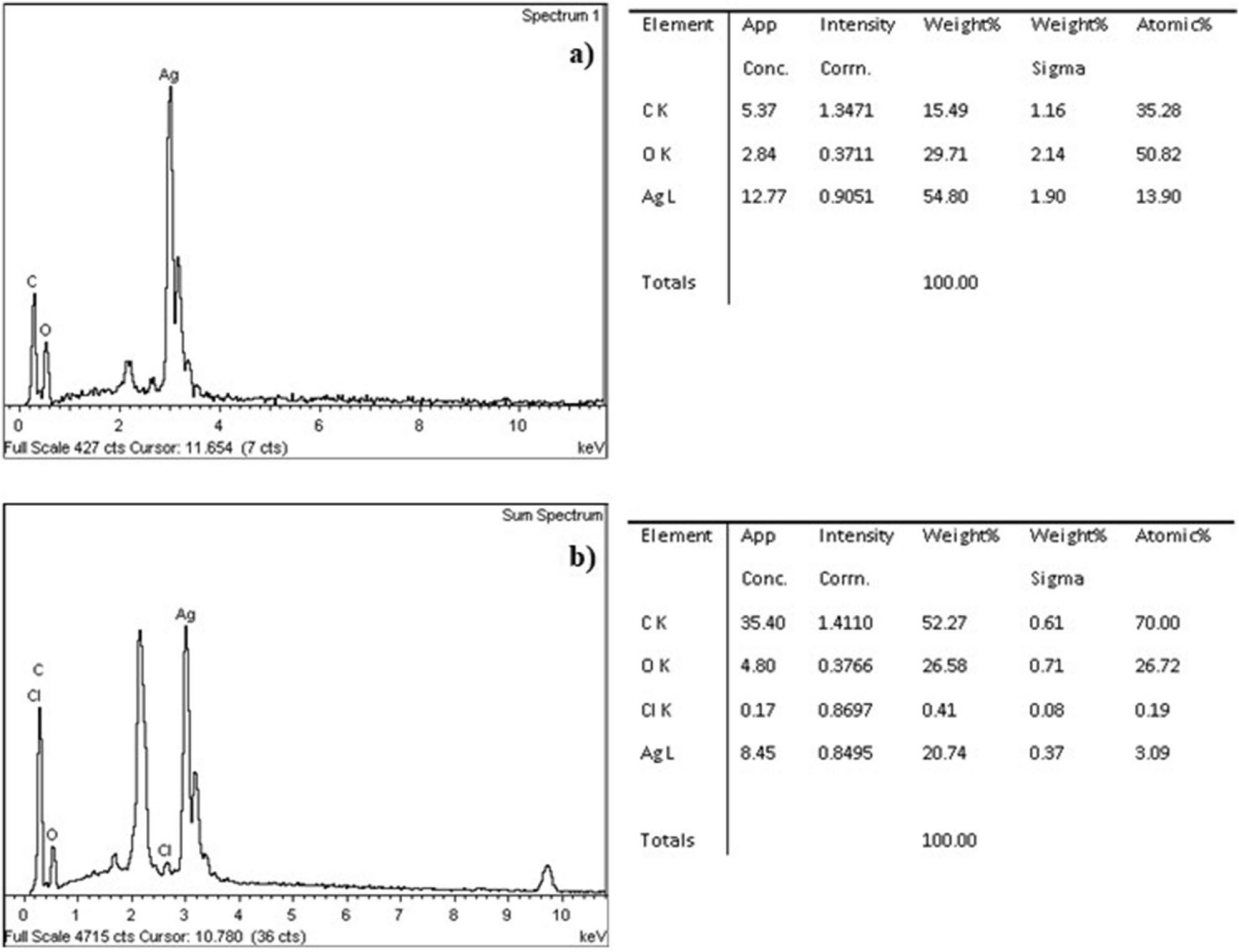
EDX spectra-data of the fungal mycelium anamorphous *Bjerkandera* sp. R1 treated with a 1 mM AgNO_3_ solution. (**a**) Growth time 5 days. 144 h of incubation time*.* (**b**) Growth time 8 days. 144 h of incubation time.

Since the best synthesis results were seen for the biomass obtained from day 8 of growth time of fungus (Fig. 11f, see black circles), it is likely that the long-time exposure caused the depletion of the carbon and nitrogen sources present in the fungal filtrate, and therefore facilitating the lysis of the fungal mycelium. In this context, the membrane becomes more permeable; therefore, more extracellular substance (proteins) is retained between the interstices of the mycelium (Fig. 12b). Under these conditions, it was possible that at the time of reaction with 1 mM AgNO_3_ there was a greater adsorption of AgNPs on the surface of the mycelium, causing a better coating and greater stabilization of particle size. The contrary occurred using biomass from shorter growth times (for example, 5 days of growth time of the fungus) since little extracellular substance accumulated in the interstitial spaces (in these conditions the fungal mycelium is not smoothed, (Fig. 12a) and the process of stabilization and reduction of AgNPs was possibly affected, generating agglomerates and macroparticles formation. The EDX support showed strong signs of Ag^0^^14,30^ and other elements such as carbon and oxygen. Regarding to these results and considering what has been reported by Taboada-Puig et al.^24^, where it is argued that growth long periods of the same fungus increase the production of proteins; it could be stated that a higher concentration of carbonyl groups (Figs. 10 and 12b) act better as a capping agent by arresting the nucleation growth of AgNPs during formation, giving rise to small sized particles (Fig. 11f, see black circles).

## Conclusions

The effect of silver nitrate (AgNO_3_) concentrations on the silver nanoparticle synthesis (AgNPs) was evaluated and was established that this factor significantly affected the behavior of the fungus anamorphous *Bjerkandera* sp. R1 against the ion reduction both in the fungal extract and on the mycelium surface. The best synthesis behavior was observed for an incubation time of 144 h using a 1 mM AgNO_3_ concentration. When evaluating the effect of growth time of fungus on the synthesis of AgNPs using this concentration, it was possible to corroborate that proteins on surface of the mycelium or chemical functional groups fell off more easily on the fungal extract from day 8, thus reducing most of the Ag^+^ in the 1 mM AgNO_3_ solution (CS sample) in Ag^0^ nanoparticles. Finally, it was found that the increase of the interstitial spaces of the mycelium was favored when the fungus grew during this period. This condition triggered greater adsorption of the silver ions on the surface of the mycelium. In this context and under prolonged reaction times with 1 mM AgNO_3_ (144 h), the greatest reduction of Ag^+^ ions in situ to Ag^0^ (MP sample) occurred.
